# Genetic analysis of resistance and virulence characteristics of clinical multidrug-resistant *Proteus mirabilis* isolates

**DOI:** 10.3389/fcimb.2023.1229194

**Published:** 2023-08-11

**Authors:** Ying Li, Ming Yin, Chengju Fang, Yu Fu, Xiaoyi Dai, Wei Zeng, Luhua Zhang

**Affiliations:** ^1^The School of Basic Medical Science and Public Center of Experimental Technology, Southwest Medical University, Luzhou, Sichuan, China; ^2^Department of Clinical Laboratory, The Hejiang People’s hospital, Luzhou, Sichuan, China

**Keywords:** *Proteus mirabilis*, ICE, blaCTX-M-65, PmGRI1, virulence

## Abstract

**Objective:**

*Proteus mirabilis* is the one of most important pathogens of catheter-associated urinary tract infections. The emergence of multidrug-resistant (MDR) *P. mirabilis* severely limits antibiotic treatments, which poses a public health risk. This study aims to investigate the resistance characteristics and virulence potential for a collection of *P. mirabilis* clinical isolates.

**Methods and results:**

Antibiotic susceptibility testing revealed fourteen MDR strains, which showed high resistance to most β-lactams and trimethoprim/sulfamethoxazole, and a lesser extent to quinolones. All the MDR strains were sensitive to carbapenems (except imipenem), ceftazidime, and amikacin, and most of them were also sensitive to aminoglycosides. The obtained MDR isolates were sequenced using an Illumina HiSeq. The core genome-based phylogenetic tree reveals the high genetic diversity of these MDR *P. mirabilis* isolates and highlights the possibility of clonal spread of them across China. Mobile genetic elements SXT/R391 ICEs were commonly (10/14) detected in these MDR *P. mirabilis* strains, whereas the presence of resistance island *Pm*GRI1 and plasmid was sporadic. All ICEs except for ICE*Pmi*Chn31006 carried abundant antimicrobial resistance genes (ARGs) in the HS4 region, including the extended-spectrum β-lactamase (ESBL) gene *bla*_CTX-M-65_. ICE*Pmi*Chn31006 contained the sole ARG *bla*_CMY-2_ and was nearly identical to the global epidemic ICEPmiJpn1. The findings highlight the important roles of ICEs in mediating the spread of ARGs in *P. mirabilis* strains. Additionally, these MDR *P. mirabilis* strains have great virulence potential as they exhibited significant virulence-related phenotypes including strong crystalline biofilm, hemolysis, urease production, and robust swarming motility, and harbored abundant virulence genes.

**Conclusion:**

In conclusion, the prevalence of MDR *P. mirabilis* with high virulence potential poses an urgent threat to public health. Intensive monitoring is needed to reduce the incidence of infections by MDR *P. mirabilis*.

## 1 Introduction

*Proteus mirabilis*, a Gram-negative rod-shaped bacterium belonging to the Morganellaceae family of the order Enterobacterales, is well-known for its urease production and characteristic bull’s-eye-pattern swarming motility on agar plates (Hamilton et al., 2018). While the bacterium can cause a variety of human infections, it is most noted as a pathogen of catheter-associated urinary tract infections (CAUTIs) (Schaffer et al., 2015). These CAUTIs frequently progress to bacteremia due to *P. mirabilis*, which carries a high mortality rate (Schaffer et al., 2015; Armbruster et al., 2018). *P. mirabilis* possesses a diverse set of virulence factors relevant to CAUTIs, such as motility, the production of urease, hemolysins, biofilm formation, adhesin, and fimbriae-mediated adherence (Armbruster et al., 2018). Once established in the catheterized urinary tract, *P. mirabilis* usually causes chronic infection and blockage that are extremely difficult to eliminate (Wasfi et al., 2020).

Owing to the flagella-mediated motility, *P. mirabilis* can easily contact uroepithelial cells, thereby promoting internalization and cytotoxicity, and facilitate transmission of the infection upward into the bladder and kidneys (Armbruster et al., 2018). *P. mirabilis* can simultaneously express multiple types of fimbriae, and several of them are implicated in virulence, such as the best characterized mannose-resistant *Proteus*-like (MR/P) fimbriae encoded by the *mrp* operon (*mrpABCDEFGHJ*) (Pearson and Mobley, 2008). Urease is a nickel-metalloenzyme that acts by hydrolyzing urea into ammonia and carbon dioxide, which is implicated in the development of infection-induced stone formation (Rutherford, 2014). CAUTIs generally stem from the formation of unusual crystalline biofilm structures on catheter surfaces (Schaffer et al., 2015). *P. mirabilis* is a biofilm former on the surface of living or abiotic surfaces and is capable to form crystalline biofilm in the urinary environment with the help of urease and adhesive proteins, such as MR/P fimbriae (Armbruster et al., 2018). The formation of extensive crystalline biofilms can occlude the urine flow through the catheter, which frequently leads to the reflux of infected urine to the kidneys and causes pyelonephritis, septicemia, and shock (Holling et al., 2014).

*Proteus* species possess intrinsic resistance to colistin, nitrofurantoin, tigecycline, and tetracycline (Girlich et al., 2020). In recent years, multidrug-resistant (MDR) *P. mirabilis* isolates are becoming increasingly common (Falagas and Karageorgopoulos, 2008; Li et al., 2022). They have been frequently described with multiple acquired antimicrobial resistance genes (ARGs) encoding extended-spectrum β-lactamases (ESBLs), such as CTX-M-65 (Lei et al., 2018b), and carbapenemases such as KPC-2 and NDM-1(Hua et al., 2020; He et al., 2021), and show co-resistance to fluoroquinolones, aminoglycosides, and sulfamethoxazole-trimethoprim (Korytny et al., 2016; Lei et al., 2018b; Shaaban et al., 2022). The prevalence of MDR *P. mirabilis* isolates and their ongoing acquisition of ARGs pose challenges to clinical treatments.

Mobile genetic elements, including plasmids, and resistance genomic islands such as integrative and conjugative elements (ICEs), play a central role in the acquisition and spread of ARGs in *P. mirabilis* (Lei et al., 2016; Li et al., 2021; Ma et al., 2021). ICE is a distinct region of a bacterial chromosome that is self-transmissible by conjugation (Partridge et al., 2018). Especially, the SXT/R391 family of ICEs constitutes a diverse group of mobile elements that carry multidrug resistance genes in *Proteus.* SXT/R391 ICEs are characterized by a conserved integrase that mediates the integration into the 5′ end of the chromosomal *prfC* gene by site-specific recombination (Wozniak et al., 2009). SXT/R391 ICEs share a conserved backbone consisting of 52 nearly identical core genes (Li et al., 2016a; Sato et al., 2020), and also contain variable DNA regions, dubbed hotspots (HS1 to HS5) and variable regions (VRI-VRV) that carry genes for antimicrobial resistance, such as *bla*_CMY-2_ (Lei et al., 2016), *tet*(X6) (He et al., 2020), and *bla*_NDM-1_ (Kong et al., 2020). SXT/R391 ICEs in *P. mirabilis* also carried multi-resistance gene *cfr* and tigecycline resistance gene cluster *tmexCD3-toprJ1* (Ma et al., 2022).

The present study was conducted to investigate the prevalence, resistance gene profiles, and virulence determinants for clinical *P. mirabilis* isolates from a county hospital in the Sichuan province of China. Also, virulence characteristics including motility, urease production, hemolytic activity, and biofilm formation were determined to better understand the potential risk of these MDR *P. mirabilis* isolates.

## 2 Materials and methods

### 2.1 Bacterial isolates

32 *P. mirabilis* strains were isolated from different clinical samples of patients at the Hejiang County People’s Hospital, in Luzhou City, Sichuan Province of China, from January to December 2021. Written informed consent from the patients was exempted from this study since the present study only focused on bacteria, and the strains were isolated as a part of the routine hospital laboratory procedures. Isolates were initially identified with both the VITEK 2 system (BioMérieux, Marcy-l’Étoile, France) and 16S rRNA gene sequencing analysis (Lane, 1991).

### 2.2 Antimicrobial susceptibility testing

The minimum inhibitory concentrations (MICs) of 23 antimicrobial agents, including ampicillin, amikacin, ampicillin/sulbactam, azlocillin, aztreonam, cefazolin, cefepime, cefoxitin, cefixime, cefotetan, ceftazidime, ceftriaxone, ciprofloxacin, ertapenem, gentamicin, imipenem, meropenem, levofloxacin, nitrofurantoin, mezlocillin, piperacillin/tazobactam, tobramycin, and trimethoprim-sulfamethoxazole were automatically performed by the VITEK 2 system. MICs of ceftazidime, cefotaxime, and meropenem were manually confirmed using the broth microdilution method, and the susceptibility testing for ceftazidime was also performed by Kirby Bauer disk diffusion method, with *Escherichia coli* strain ATCC 25922 as the quality control. The results of antibiotic susceptibility testing were interpreted by the breakpoints defined by the Clinical and Laboratory Standards Institute standards for Enterobacterales (CLSI, M100) (CLSI, 2023). MDR strains were defined as non-susceptibility to three or more of the following antibiotic groups: β-lactam-β-lactam inhibitor combinations, cephalosporins, aminoglycosides, fluoroquinolones or trimethoprim-sulfamethoxazole (Korytny et al., 2016).

### 2.3 Genomic sequencing and bioinformatic analysis

Fourteen MDR *P. mirabilis* were selected for genomic DNA extraction using the QIAamp DNA Mini Kit (Qiagen, Hilden, Germany) following the manufacturer’s guidelines. Whole genome sequencing was performed on the HiSeq 2000 (Illumina, San Diego, CA, USA) Sequencer with a 150 bp paired-end library and 200 × coverage by the Beijing Tsingke Bioinformatics Technology Co. Ltd. The raw reads were trimmed using Trimmomatic v0.38 before being assembled into draft genomes using SPAdes v3.12.0 program (Bankevich et al., 2012; Bolger et al., 2014). Annotation was carried out using Prokka (Seemann, 2014). Genome-based species identification was performed by average nucleotide identity (ANI) analysis. The ANI value between genome sequences of these clinical isolates and that of reference strain *P. mirabilis* HI4320 (Accession no. NC_010554) was calculated with JSpeciesWS (Richter et al., 2016). 96% is the cut-off for defining a bacterial species. Plasmid incompatibility types, antibiotic resistance genes (ARGs), and insertion elements (ISs) were predicted using PlasmidFinder (Carattoli and Hasman, 2020), ResFinder (Bortolaia et al., 2020), and ISfinder (Siguier et al., 2006). The detection of SXT/R391 ICE in the whole genome sequences was performed using the conserved integrase gene (*int*_SXT_). The contigs of SXT/R391 ICE were extracted and assembled against the reference ICE of FZP3105 from our previous study (Li et al., 2022), and gaps between contigs were closed by PCR and Sanger sequencing. The contigs of *Pm*GRI1 were extracted and assembled against the reference *Pm*GRI1-CYPM1 (GenBank accession CP012674). Linear sequence alignment was carried out using BLAST and visualized with Easyfig 2.2.3(Sullivan et al., 2011).

### 2.4 Phylogenetic analysis

The genome sequences of other representative *Proteus* isolates in China were retrieved from the GenBank. Genetic relationship of different *Proteus* isolates was assessed based on single nucleotide polymorphisms (SNPs) in their core genomes, as previously described with minor modification (Li et al., 2022). Briefly, the GFF3 files generated by Prokka were piped into Roary to create a core genome alignment. SNPs were extracted using snp-sites v2.3.2 (Kwok and Hitchins, 2015). A maximum-likelihood phylogenetic tree was constructed based on the SNPs using FastTree version 2.1.10 under the GTRGAMMA model with 1000 bootstrap iterations (Price et al., 2010).

### 2.5 Transferability assay

Conjugation experiments were carried out using broth-based method with the *E. coli* strain J53 (sodium azide-resistant) or EC600 (rifampicin-resistant) as the recipient, as described previously with minor modification (Li et al., 2022). After the donor and recipient strains were grown to exponential stage (the optical density at 600 nm reaches ~0.5), mix them at a ratio of 1:1 before incubation at 37°C for 24 h. Transconjugants were selected on Luria-Bertani (LB) agar plates containing 4 μg/ml cefotaxime plus 150 μg/ml sodium azide (for J53) or 200 μg/ml rifampicin (for EC600). The presence of SXT/R391 ICE was confirmed by PCR using the primers targeting *int*_SXT_ (Sato et al., 2020).

### 2.6 Measurement of virulence

#### 2.6.1 Crystalline biofilm assay

Broth cultures of *P. mirabilis* (OD600~0.4) were diluted 1/100 into fresh LB broth containing 50% filter sterilized human urine prepared from a pool of urine from several healthy volunteers. 200 µl diluted bacterial cultures were incubated statically in sterile 96-well microtiter plates (Costar 3599, Corning, NY, USA) for 24 h at 37°C. The culture supernatant was then removed and the biofilm production was quantified by the crystal violet staining method as described previously(Li et al., 2016b). Negative control wells contained only LB broth and human urine. The average OD595 values were calculated for sextuplicate, and the tests were repeated three times. The cut-off value (ODc) was defined as 3 SD above the mean OD595 of the negative control. If OD595≤ODc, absence of biofilm; if ODc≤OD595 ≤ 2×ODc, weak biofilm producer; if 2×ODc≤OD595 ≤ 4×ODc, moderate biofilm producer; if 4×ODc≤OD595, strong biofilm producer(Qu et al., 2022).

#### 2.6.2 Urease, hemolysis, and motility assays

The urease quantification assay was performed as described previously (Durgadevi et al., 2019a). Briefly, overnight culture of *P. mirabilis* was mixed with LB broth containing filter sterilized urea (20 g/L) in the ratio of 1:100. After incubation for 24 h at 37°C, the color change (orange to pink) was observed by adding 0.02% phenol red reagent (pH indicator). The hemolytic ability of *P. mirabilis* strains was determined by culturing on a 10% sheep blood plate for 24 h at 37 °C. For motility assays, 1 µl of culture was point inoculated onto the surface center of the solid LB agar plates. After incubation for 24 h at 37 °C in a lid-side-up position, motility was measured as the diameter across which *P. mirabilis* grew (Durgadevi et al., 2019b).

### 2.7 Virulence genes analysis

The presence of genes related to bacterial virulence factors including fimbriae (*mrpA*, *ucaA*, *pmfA*, *pmpA*), hemolysin (*hpmAB*), Urease (*ureC*), biofilm formation (*pst*, *rcsD*), autotransporters (*pta*, *aipA*), proteases (*zapA*), and siderophore (*nrpR*) were identified using the Virulence Factors of Pathogenic Bacteria Database (VFDB) (Chen et al., 2005). The identification of flagella genes (*flhA*, *fliF*, *fliG*, *fliP*, *fliL*, *flgN*, *flaD*) was performed in a pairwise BLASTn alignment.

## 3 Results and discussion

### 3.1 Sources and antimicrobial susceptibility profiles of MDR *P. mirabilis* strains

Among the 32 P*. mirabilis* strains, 14 (43.8%) of them were identified as MDR, which were recovered from sputum (n=7, 50%), urine (n=4, 28.6%), stool (n=1, 7.1%), ascites (n=1, 7.1%), and wound secretion (n=1, 7.1%). Results of the antimicrobial susceptibility test indicated that all the MDR strains were resistant to ampicillin, azlocillin, cefazolin, cefixime, mezlocillin, ampicillin/sulbactam, and trimethoprim/sulfamethoxazole, in addition to their intrinsic resistance profiles (**Table 1**). They also showed high resistance rates (>70.0%) to ceftriaxone (n =12, 85.7%), and a lesser extent to cefotetan (n =8, 57.1%), ciprofloxacin (n=8, 57.1%), and levofloxacin (n=8, 57.1%). Four isolates (28.6%) were resistant to piperacillin/tazobactam. Resistance to antimicrobial agents, including cefepime, cefoxitin, aztreonam, gentamicin, and tobramycin was rarely (n<3) detected. All the MDR strains were sensitive to meropenem (MIC ≤ 1), ertapenem (MIC ≤ 0.5), ceftazidime (MIC ≤ 1), and amikacin (MIC ≤ 2). The Kirby Bauer test confirmed the sensitivity of these strains to ceftazidime. Consistent with the observation that *P. mirabilis* possesses intrinsic decreased susceptibility to imipenem (but not meropenem and ertapenem), 92.9% of MDR strains (n=13) were resistant to imipenem in this study, which was thought to be caused by weak affinity with penicillin-binding proteins or porin loss (Girlich et al., 2020).

**Table 1:** Clinical information and genomic characterization of 14 MDR *P. mirabilis* isolates.

Strain	Specimen	Age (Year) /Gender^a^	Antimicrobial resistance genes	Resistance phenotype^b^	Plasmid replicons	Genomic islands
HJP21048	Wound secretion	72/M	*aac(6’)-Ib-cr*, *aph(3’)-Ia*, *aadA2b*, *arr-3*, *bleO*, *cat*, *catB3*, *fosA3*, *ere*(A), *tet*(C), *tet*(J), *bla*_OXA-1_, *qnrD1*, *sul1*, *dfrA32*	AMP, AZL, SAM, CZO, CEF, CTT, IPM, MEZ, NIT, SXT	Col3M	ICE
HJP21049	Ascites	93/F	*aac(6’)-Ib-cr*, *aph(4)-Ia*, *aadA2b*, *aac(3)-IV*, *arr-3*, *dfrA32*, *bleO, bla*_OXA-1_, *bla*_CTX-M-65_, *fosA3*, *qnrD1*, *catB3*, *floR*, *ere*(A), *tet*(C), *tet*(J), *sul1*, *sul2*	AMP, AZL, SAM, CZO, CEF, CRO, CIP, IPM, LVX, MEZ, NIT, SXT	Col3M	ICE
HJP16012	Stool	72/M	*aac(3)-IIa*, *aph(6)-Id*, *aph(3’’)-Ib*, *aph(3’)-Ia*, *aadA5*, *mph*(A), *catA1*, *cat*, *dfrA17*, *tet*(J), *bla*_TEM-1B_, *sul1*, *sul2*	AMP, AZL, SAM, CZO, CEF, CTT, CIP, IPM, LVX, MEZ, NIT, SXT	IncQ1	
HJP22021	Urine	71/M	*aac(6’)-Ib-cr*, *aadA1*, *aph(4)-Ia*, *aac(3)-IV*, *dfrA1*, *arr-3*, *bla*_OXA-1_, *bla*_CTX-M-65_, *cat*, *floR*, *catA1*, *catB3*, *tet*(J), *fosA3*, *sul1*, *sul2*	AMP, AZL, SAM, CZO, CEF, CTT, CRO, CIP, IPM, LVX, MEZ, NIT, TZP, SXT		*Pm*GRI1
HJP05004	Urine	53/M	*aac(6’)-Ib-cr*, *aadA1*, *aac(3)-IV*, *aph(6)-Id*, *aadA5*, *aph(3’’)-Ib, aph(4)-Ia, aadA2b, aph(3’)-Ia*, *aac(3)-IId*, *bla*_OXA-1_, *bla*_CTX-M-63_, *bla*_TEM-1B_, *arr-3*, *catA1*, *catB3*, *cat*, *cmlA1*, *floR*, *dfrA17*, *dfrA1*, *tet*(J), *sul1*, *sul2*	AMP, AZL, SAM, CZO, CEF, CTT, CRO, CIP, GEN, IPM, LVX, MEZ, NIT, SXT	IncQ1	*Pm*GRI1
HJP18031	Sputum	89/M	*catA1*, *cat*, *floR*, *qnrS1*, *tet*(J)	AMP, AZL, SAM, CZO, CEF, CTT, CRO, CIP, IPM, LVX, MEZ, NIT, TOB, SXT		
HJP17012	Sputum	75/M	*aph(3’)-VIa, aadA1, aph(3’’)-Ib, aph(6)-Id, aadA2b, aac(6’)-Ib-cr*, *aph(4)-Ia*, *aac(3)-IV*, *arr-3*, *bla*_OXA-1_, *bla*_CTX-M-65_, *bla*_TEM-1B_, *tet*(J), *floR*, *fosA3*, *cmlA1*, *cat*, *catB3*, *catA1*, *dfrA1*, *sul1*, *sul2*, *sul3*	AMP, AZL, SAM, CZO, CEF, CTT, FEP, CRO, CIP, IPM, LVX, MEZ, NIT, TZP, SXT		ICE, *Pm*GRI1
HJP20004	Sputum	81/M	*aac(6’)-Ib-cr*, *aph(6)-Id, aph(3’’)-Ib*, *aadA2b*, *aph(4)-Ia, aac(3)-IV*, *aph(3’)-Ia, aadA1, dfrA1*, *dfrA32*, *ere*(A), *tet*(J), *tet*(C), *arr-3*, *bla*_OXA-1_, *bla*_CTX-M-65_, *cat*, *catA1*, *catB3*, *floR*, *sul1*, *sul2*	AMP, AZL, SAM, CZO, CEF, CRO, CIP, IPM, MEZ, NIT, SXT		ICE, *Pm*GRI1
HJP31006	Vaginal secretion	28/F	*aph(4)-Ia*, *aadA2*, *aac(3)-IV*, *aph(3’)-Ia, bleO*, *bla*_CMY-2_, *dfrA12*, *qnrD1*, *tet*(J), *floR*, *cat*, *sul2*	AMP, AZL, SAM, CZO, CEF, CTT, CIP, IPM, LVX, MEZ, NIT, TZP, SXT	Col3M	ICE
HJP22016	Urine	57/F	*aadA2b*, *aph(4)-Ia*, *aac(3)-IV*, *bla*_CTX-M-65_, *ere*(A), *fosA3*, *qnrD1*, *dfrA32*, *dfrA1*, *cat*, *floR*, *catB3*, *tet*(J), *tet*(C), *sul2*	AMP, AZL, SAM, ATM, CZO, CEF, CTT, FEP, CRO, IPM, LVX, MEZ, NIT, TZP, SXT	Col3M	ICE, *Pm*GRI1
HJP26021	Sputum	78/M	*aph(3’’)-Ib*, *aac(3)-IV*, *aadA2b, aph(6)-Id*, *aac(6’)-Ib-cr, aph(4)-Ia, aph(3’)-VIa*, *arr-3*, *bla*_OXA-1_, *bla*_CTX-M-65_, *catB3*, *cat, dfrA32*, *ere*(A), *floR, fosA3, qnrD1, tet*(C), *tet*(J), *sul1*, *sul2*	AMP, AZL, SAM, CZO, CEF, CRO, IPM, MEZ, NIT, SXT	Col3M	ICE
HJP31010	Sputum	78/M	*aph(3’’)-Ib*, *aph(4)-Ia, aadA2b, aac(3)-IV*, *aph(3’)-VIa, aph(6)-Id, aac(6’)-Ib-cr*, *arr-3*, *bla*_OXA-1_, *bla*_CTX-M-65_, *catB3*, *cat*, *dfrA32*, *ere*(A), *floR*, *fosA3*, *qnrD1, tet*(C), *tet*(J), *sul1*, *sul2*	AMP, AZL, SAM, CZO, CEF, CRO, IPM, MEZ, NIT, SXT	Col3M	ICE
HJP31030	Urine	80/M	*aph(3’’)-Ib*, *aac(3)-IV*, *aadA2b, aph(6)-Id*, *aph(3’)-VIa*, *aph(4)-Ia*, *aac(6’)-Ib-cr*, *arr-3, bla*_OXA-1_, *bla*_CTX-M-65_, *catB3*, *cat, dfrA32*, *ere(A)*, *floR*, *fosA3, qnrD1*, *tet*(C), *tet*(J), *sul1*, *sul2*	AMP, AZL, SAM, CZO, CEF, CRO, MEZ, NIT, SXT	Col3M	ICE
HJP01027	Sputum	78/M	*aac(6’)-Ib-cr*, *aph(4)-Ia, aph(3’’)-Ib*, *aadA2b*, *aph(3’)-VIa*, *aac(3)-IV, aph(6)-Id, arr-3, bla*_OXA-1_, *bla*_CTX-M-65_, *catB3*, *cat*, *dfrA32*, *ere*(A), *floR*, *fosA3*, *qnrD1*, *tet*(C), *tet*(J), *sul1*, *sul2*	AMP, AZL, SAM, CZO, CEF, CRO, IPM, MEZ, NIT, SXT	Col3M	ICE

a Gender: M, male; F, female.

b AMP, ampicillin; ATM, aztreonam; AZL, azlocillin; CIP, ciprofloxacin; CRO, ceftriaxone; CTT, cefotetan; CEF, cefixime; CZO, cefazolin; FEP, cefepime; FOX, cefoxitin; GEN, gentamicin; IPM, imipenem; LVX, levofloxacin; MEZ, mezlocillin; NIT, nitrofurantoin; SAM, ampicillin/sulbactam; SXT, trimethoprim/sulfamethoxazole; TOB, tobramycin; TZP, piperacillin/tazobactam.

### 3.2 Genotypic resistance of MDR *P. mirabilis* strains

We sequenced the genomes of all 14 MDR isolates on the Illumina platform. The genomes vary from 3,964,688 bp to 4,212,070 bp, with an average GC content of 38.80% to 39.09% (**Table S1**). The genomic analysis identified the presence of 35 different ARGs in the 14 MDR *P. mirabilis* strains, and 11 (78.6%) of them carried at least 15 ARGs (**Table 1**). Three different ESBLs genes, *bla*_CTX-M-65_, *bla*_CTX-M-63_, and *bla*_CMY-2_, were detected. Among them, *bla*_CTX-M-65_, which has been identified in many *P. mirabilis* strains from humans and animals in China (Li et al., 2022; Qu et al., 2022), also represents the most prevalent one in this study. All the strains except for HJP18031 carried abundant aminoglycosides resistance genes, with *aac(3)-IV* (n=11), *aph(4)-Ia* (n=11), *aph(6)-Id* (n=8), and *aph(3’’)-Ib* (n=8) being the most prevalent. Of the detected quinolone resistance genes, *aac(6’)-Ib-cr* (n=10, 71.4%) is the most common, followed by *qnrD1* (n=8, 57.1%), and *qnrS1* was only detected in one isolate. Furthermore, we found that genotypic and phenotypic resistance in these strains often does not match. Almost all the *bla*_CTX-M-65_-harboring isolates (except for HJP22016) remain sensitive to aztreonam. Also, the presence of *aac(6’)-Ib-cr* did not lead to quinolone resistance in several strains, such as HJP21048, HJP31010, and HJP31030. These findings suggest that the functions of these ARGs may be regulated by some unknown mechanisms in *P. mirabilis* strains (Li et al., 2022).

### 3.3 Genomic phylogeny of MDR *P. mirabilis* strains

**Figure 1:**
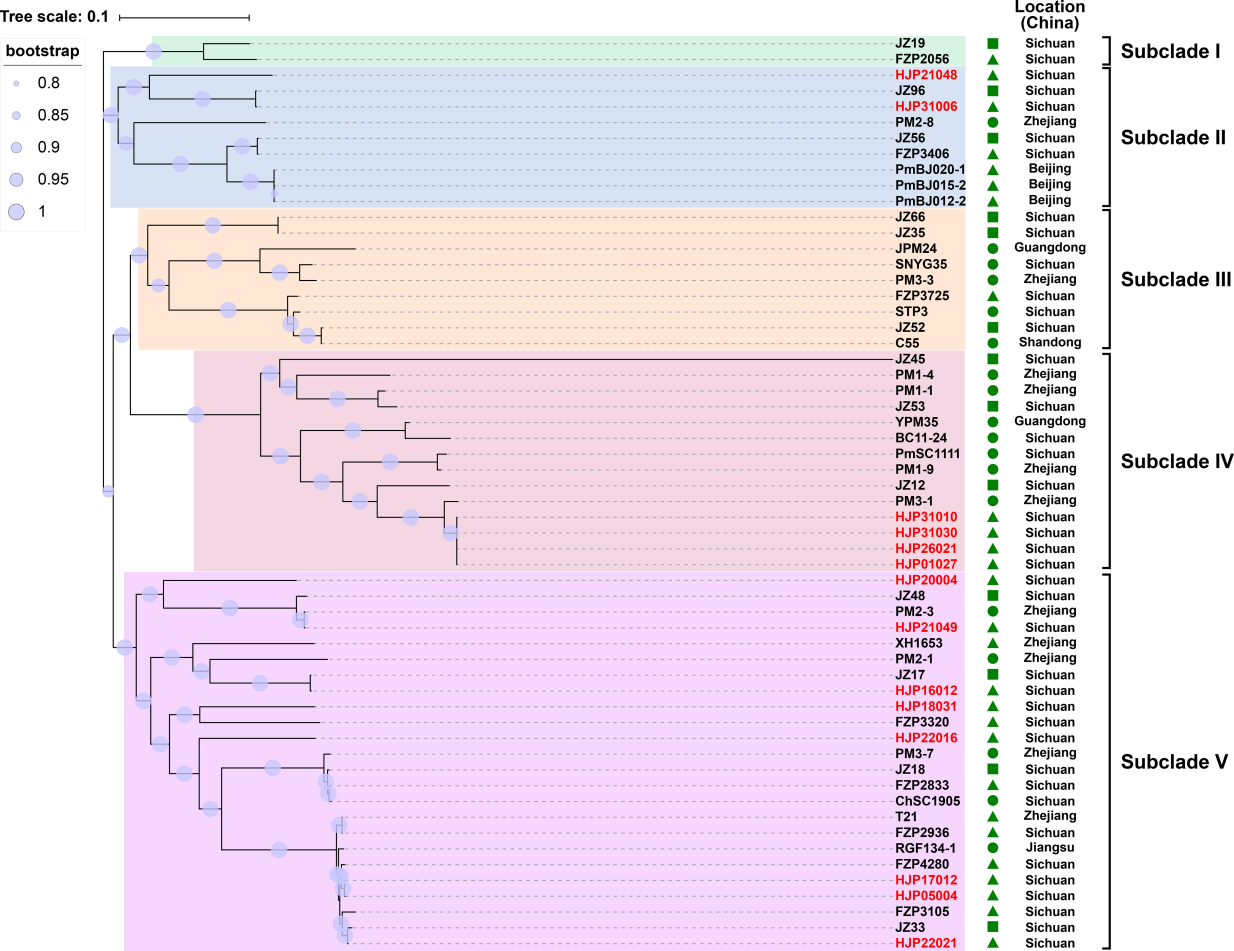
Phylogenetic analysis of MDR *P. mirabilis* strains. The phylogenetic tree was constructed based on the SNPs in their core genome with the maximum-likelihood method in FastTree. Strains identified in this study are highlighted in red. The host of the isolate is indicated by triangles (animals), squares (food) or circles (humans). The tree scale indicates substitutions per site.

To understand the genetic relationship of 14 MDR *P. mirabilis* strains in this study with other isolates from different geographic locations in China (**Table S2**), a core genome-based phylogenetic tree was constructed, which revealed five distinct groups (**Figure 1**). The tested strains are diffusely distributed in the tree, suggesting the genetic diversity of them. HJP21048 and HJP31006 are present in the subclade II that is distant from other tested strains, suggesting a different origin of them. In subclade IV, four strains HJP26021, HJP31010, HJP31030, and HJP01027 are tightly clustered, and they share identical core genomes (SNP=0) as well as resistance phenotypes, showing the clonal nature of them. Notably, HJP26021, HJP31010, and HJP01027 were subsequent isolates from the sputum of a single patient a few days apart, whereas HJP31030 was from another patient in the same inpatient ward but a different room, indicating the clone spread of this MDR strain. In subclade V, strains from this study cluster with several isolates from animals and humans from different locations in China. Especially, HJP17012 and HJP05004 are closely related (105 SNPs), and tightly cluster together with several clinical isolates and animal-derived strains. These findings suggest the possibility of circulation and clonal spread of these MDR *P. mirabilis* strains across different regions of China.

### 3.4 Genetic features of ARG-bearing plasmids in MDR *P. mirabilis* strains

**Figure 2:**
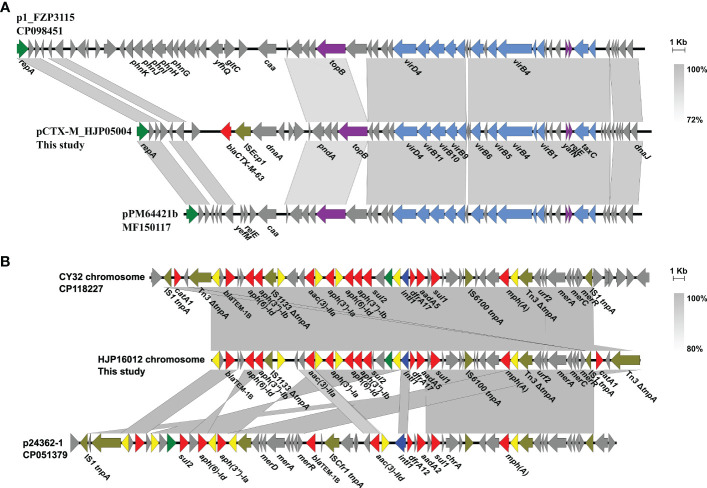
Genetic organization of plasmids or plasmid-derived region in MDR *P. mirabilis* strains. **(A)** Comparison of pCTX-M_HJP05004 with p1_FZP3115. Genes are denoted by arrows. *bla*_CTX-M-63_, IS*Ecp1*, *repA*, conjugation genes, and maintenance genes are highlighted in red, olive, green, blue, and purple, respectively. Regions of >73% identity are indicated by grey shading. **(B)** Organization of the plasmid-derived region on the chromosome of HJP16012, and comparisons to related regions. Genes are denoted by arrows. ARGs, replication genes, integrase genes, *IS26*, and other transposase genes are highlighted in red, green, blue, yellow, and olive, respectively. Regions of > 80% identity are indicated by grey shading. Δ represents truncated genes.

Only two types of plasmids, IncQ1 and Col3M, were identified in these *P. mirabilis* strains and eight of them harbor the 2,683-bp Col3M plasmid, which encodes a quinolone resistance protein QnrD1 and a hypothetical protein. BLASTn analysis showed that the Col3M plasmid is very common in *P. mirabilis* strains of different origins. Two IncQ1-type replication genes were detected in HJP05004 and HJP16012, respectively. In HJP05004, the IncQ1 plasmid pCTX-M_HJP05004 is 40,486 bp in size and has no other known ARGs except *bla*_CTX-M-63_ (**Figure 2A**). It consists of a 38-kb backbone comprising genes for replication (*repA*), maintenance (*topB*), and conjugation (*vir* genes). The sole acquired resistance gene region was identified downstream of *dnaA* (encoding the chromosomal replication initiation ATPase DnaA), wherein *bla*_CTX-M-63_ was present in an IS*Ecp1*-*bla*_CTX-M-63_ transposition unit. The insertion sequence IS*Ecp1* is known to mobilize adjacent sequences, including *bla*_CTX-M_ genes, by using its own left inverted repeat (IRL) in conjunction with alternative sequences resembling its right inverted repeat (IRR) (Zong et al., 2010). BLASTn revealed that pCTX-M_HJP05004 is partly similar (71% coverage, >97.1% nucleotide identity) to p1_FZP3115 (CP098451, *P. mirabilis*, patient) from China, and pPM64421b (MF150117, *P. mirabilis*, unknown) from Brazil, indicating global spread of this type of plasmid.

On the chromosome of HJP16012, thirteen ARGs conferring resistance to aminoglycosides (*aadA5*, *aph(6)-Id*, *aph(3’’)-Ib*, *aph(3’)-Ia*, *aac(3)-IIa*, *aph(6)-Id*, *aph(3’’)-Ib*), β-lactam (*bla*_TEM-1B_), macrolide (*mph*(A)), amphenicol (*catA1*), sulphonamides (*sul1*, *sul2*), and trimethoprim (*dfrA17*) are clustered in a 39, 086-bp MDR region, wherein an IncQ1-type replication gene *repA* is present. The MDR region showed high similarity (98% coverage, 99.56% identity) to the one found in a *P. mirabilis* strain CY32 (CP118227, chicken, China), and was partly similar (85% coverage, 99.87% identity) to the plasmid p24362-1 (CP051379) recovering from a *Salmonella enterica* strain from swine in the USA. The result reveals that the MDR regions on the chromosomes of *P. mirabilis* HJP16012 and CY32 seem to be derived from a p24362-1-like plasmid. Further sequence analysis showed that two copies of IS*1* flanked the MDR region in CY32 and HJP16012 (**Figure 2B**), suggesting a possible role of IS*1*-mediated composite transposon in the integration process (Partridge et al., 2018).

### 3.5 The resistance genomic islands in MDR *P. mirabilis* strains

In recent years, five *Salmonella* genomic island 1 (SGI1)-relative elements, including SGI1(Ahmed et al., 2006), *Proteus* genomic island 1 (PGI1)(Siebor and Neuwirth, 2014), *Proteus* genomic island 2 (PGI2)(Lei et al., 2018a), GI*Pmi*1(Siebor et al., 2018), and *Pm*GRI1(Lei et al., 2020) have been identified in *P. mirabilis* strains. The presence of SGI-relative elements in our *P. mirabilis* isolates was screened by searching the integrase gene of SGI1 (KM234279), PGI1(KJ411925), PGI2 (MG201402), GI*Pmi*1 (MF490433), and *Pm*GRI1(MW699445) from the whole sequenced genomes. As a result, five (35.71%, 5/14) strains contained the *Pm*GRI1 integrase gene (**Table 1**). No other integrase genes of SGI1-relative elements were detected in these strains.

**Figure 3:**
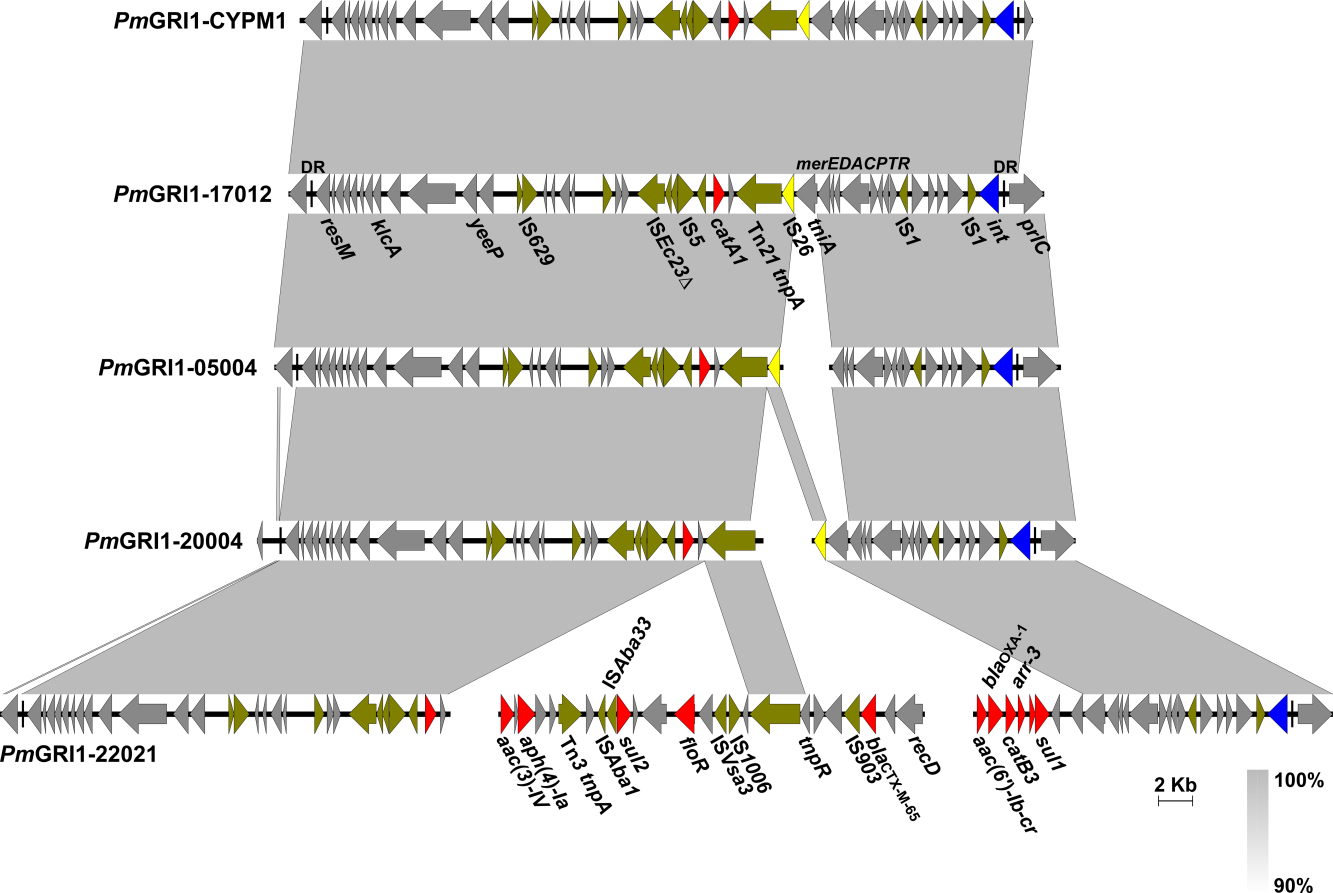
Comparative analysis of the *Pm*GRI1 in MDR *P. mirabilis* strains. Genes are denoted by arrows and the direction of transcription is indicated by the arrowheads. ARGs, integrase genes, *IS26*, and other transposase genes are highlighted in red, blue, yellow, and olive, respectively. Regions of >90% identity are indicated by grey shading. Δ represents truncated genes.

*Pm*GRI1 is a multidrug-resistant GI that has been found in several *P. mirabilis* isolates of animal and human origins from different locations in China (Ma et al., 2021; Li et al., 2022). Based on the genome data, the complete sequences of *Pm*GRI1 in strain HJP17012 (*Pm*GRI1-17012) was successfully assembled. *Pm*GRI1-17012 is 40,678 bp in size, and *catA1* is the only known and complete antimicrobial resistance gene (**Figure 3**). BLASTn analysis showed that the configuration of *Pm*GRI1-17012 is identical to that of *Pm*GRI1-CYPM1 (CP012674), which was recovered from a clinical *P. mirabilis* strain from Taiwan, China, in 2012. Due to possible complex structures or high numbers of transposases and ISs, the complete sequences of *Pm*GRI1 in HJP05004, HJP22021, and HJP20004 were fragmented in two or more contigs. *Pm*GRI1-05004 and *Pm*GRI1-20004 are also possible variants of *Pm*GRI1-CYPM1 as revealed by the existing assembly. *Pm*GRI1-22021 seems to have a more complex genetic structure, with a number of genes, including several ARGs, being inserted upstream and downstream of *tnpA* of Tn21, respectively (**Figure 3**). Altogether, the presence of *Pm*GRI1 in the tested strains reconfirmed its prevalence in *P. mirabilis* in China, highlighting that *Pm*GRI1 may serve as an important vehicle in capturing and spreading ARGs.

In addition, we detected the presence of SXT/R391 ICEs by targeting the conserved integrase gene (*int*_SXT_) and found that ten out of the fourteen (71.4%, 10/14) MDR *P. mirabilis* strains were positive for the *int*_SXT_ gene (> 96% identity to that of SXT, AY055428, **Table 1**). The complete sequences of seven SXT/R391 ICEs were successfully assembled, ranging in size from 91,092 bp to 131,933 bp. Among them, ICE*Pmi*Chn26021, ICE*Pmi*Chn31010, ICE*Pmi*Chn31030, and ICE*Pmi*Chn01027 are almost identical, mainly differing by an insertion or deletion of the *aph(6)-Id* gene (an aminoglycosides resistance gene). BLASTn analysis showed that ICE*Pmi*Chn21049 is similar (94% coverage, 99.96% nucleotide identity) to ICE*Pmi*Chn-HERJC4 (MZ221994) recovering from *P. mirabilis* strain HERJC4 from chicken in China. ICE*Pmi*Chn20004 is closely related (99% coverage, >99.9% identity) to several ICEs of human and animal origins from different locations in China, such as those in *P. mirabilis* strain PM8762 (CP092652, patient, 2021), and *P. mirabilis* strain HBNNC12 (MZ277865, cow, 2018). ICE*Pmi*Chn31006 is nearly identical (99% query coverage, 99.95% identity) to the element ICEPmiJpn1 initially described in Japan (Harada et al., 2010) and was later detected in other parts of the world (Sato et al., 2020), confirming the global spread of this element. The four clonally related ICEs, ICE*Pmi*Chn26021, ICE*Pmi*Chn31010, ICE*Pmi*Chn31030, and ICE*Pmi*Chn01027, showed the highest similarity (94% coverage, 99.95% nucleotide identity) to ICE*Pmi*Chn3105 (Accession no. CP098444), while HJP31010 (or HJP31030, or HJP01027, or HJP26021) is genetically distant from FZP3105 according to the species tree (**Figure 1**). The results suggest the cryptic dissemination and independent acquisition of MDR ICEs in *P. mirabilis* strains.

### 3.6 Genetic features of ICEs in MDR *P. mirabilis* strains

**Figure 4:**
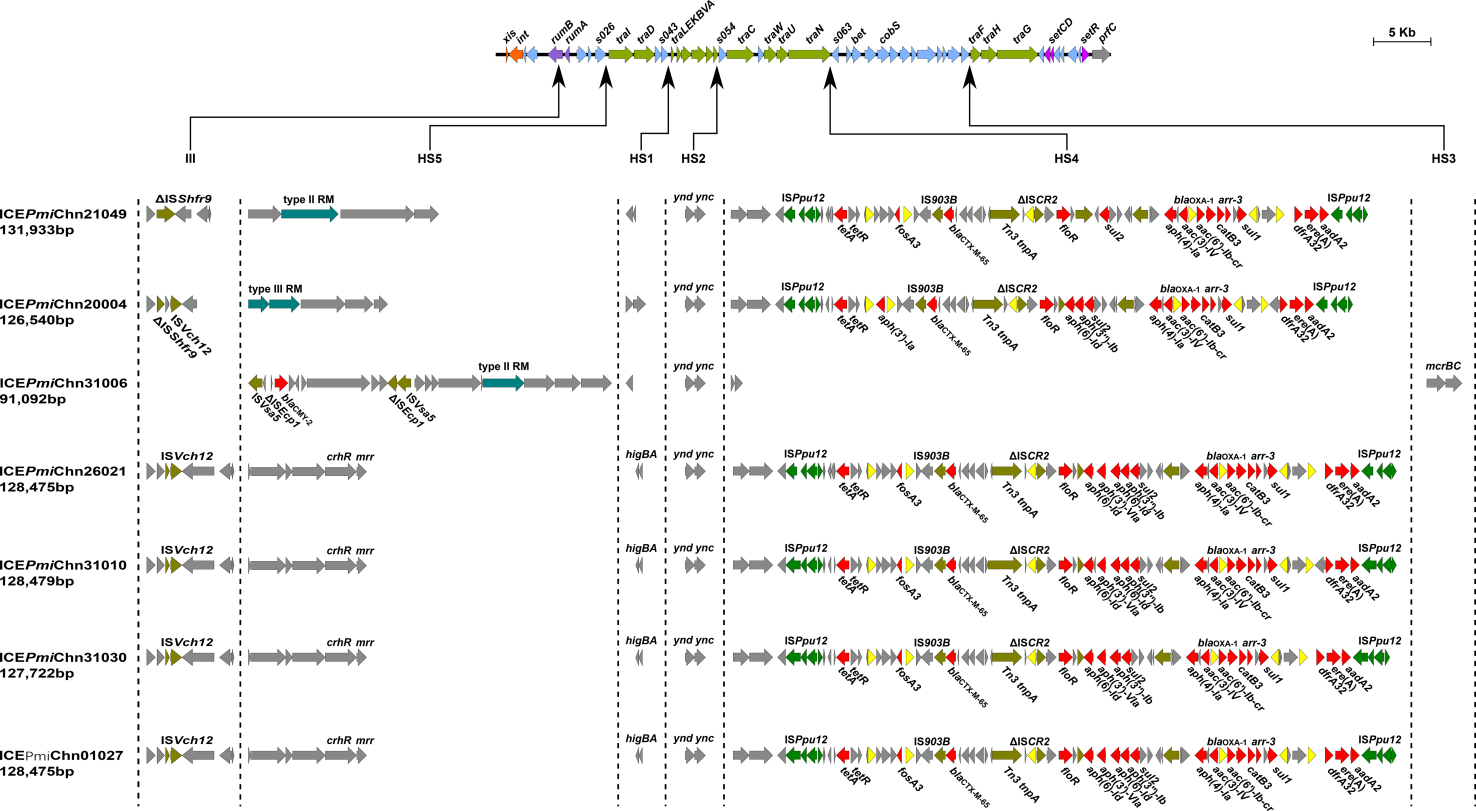
Genetic features of the ICEs in MDR *P. mirabilis* strains. The upper line shows the backbone of SXT/R391 ICEs with conserved core genes. Genes are denoted by arrows and those involved in site-specific excision and integration (*xis* and *int*), DNA repair (*rumAB*), conjugative transfer (*tra*), and regulation (*setCDR*) are highlighted in orange, purple, green, and amaranth. Under the backbone, hotspots (HS1-HS5), and variable regions (II-IV) are shown, with thin arrows indicating the sites of their insertion. Other genes in the insertion regions are indicated by light blue arrows. Δ represents truncated genes.

Genetic analysis revealed that these ICEs shared a common backbone structure with most SXT/R391 ICEs (**Figure 4**). Additionally, all ICEs except for ICE*Pmi*Chn31006 contained four hotspots (HS1, HS2, HS4, HS5) and one variable region (VR) III. Their HS4 (*traN*-*s063*) regions contain 15 to 19 ARGs (encoding for β-lactam, aminoglycoside, fluoroquinolone, fosfomycin, tetracycline, macrolide, sulphonamide, trimethoprim, and rifamycin resistance) that are clustered together in an IS*Ppu12*-mediated composite transposon (**Figure 4**). The MDR HS4 regions identified in this study are highly similar to each other, with IS*26*-mediated excision/replacement upstream of *tet*(A) and IS*CR2*-mediated insertion of ARGs downstream of *floR* representing two major modular differences of them (**Figure S1**). These HS4 regions are also similar to that in ICE*Pmi*ChnHBSZC16 (MZ277866, *P. mirabilis*, animal, China), ICE*Eco*ChnXH1815 (CP069386, *E. coli*, patient, China), and ICE*Kpn*ChnQD23 (CP042858, *Klebsiella pneumoniae*, patient, China), suggesting that ICEs may sever as an important vehicle in mediating the accumulation and dissemination of ARGs across bacterial species from different sources. ICE*Pmi*Chn31006 carries no VRIII and MDR HS4, but an HS3 region, with inserted genes encoding a pair of methylcytosine-specific restriction enzyme *mcrBC*. The sole ARG *bla*_CMY-2_ (encoding β-lactam resistance) in ICE*Pmi*Chn31006 is present in the HS5 region and locates downstream of the truncated IS*Ecp1*, the element assumed to be involved in mobilization and expression of *bla*_CMY-2_ (**Figure 4**) (Harada et al., 2010). To determine the transfer ability of these ICEs, we selected four ICE-carrying strains (HJP21049, HJP17012, HJP31006, and HJP26021) for the conjugation experiments. However, no transconjugants were obtained after repeated attempts, despite that conjugative genes were complete. The unsuccessful conjugation might result from the exceptionally low transferability that is below detectable limits.

It has been known that SXT/R391 ICEs have the potential to be transferred and thus are important agents in the dissemination of antimicrobial resistance. SXT/R391 ICEs are commonly detected in *Proteus*, as the vehicle of various resistance genes, including several clinically important ARGs, such as *bla*_CTX-M-65_ (Li et al., 2022), *bla*_NDM-1_ (He et al., 2021), and *tet*(X6) (Peng et al., 2020). The findings in this study demonstrates highly genetic plasticity in the MDR HS4 region of ICEs carried by *Proteus* strains and further highlights them as a public health threat that requires continuous monitoring.

### 3.7 Virulence characteristics of MDR *P. mirabilis* strains

All the tested strains produce strong crystalline biofilm in the urine environment and all of them, except for HJP16012, are urease producers (**Figures S2**, **S3**). The hemolysis was observed in seven strains, including HJP21049, HJP05004, HJP17012, HJP20004, HJP26021, HJP16012, and HJP31030 (**Figure S4**). In addition, different degrees of swarming migration on agar surfaces, with diameter lengths of 42 to 80 mm, were observed in these strains (**Figure S5**). Whether motility is positively correlated with bacterial virulence needs further verification.

**Figure 5:**
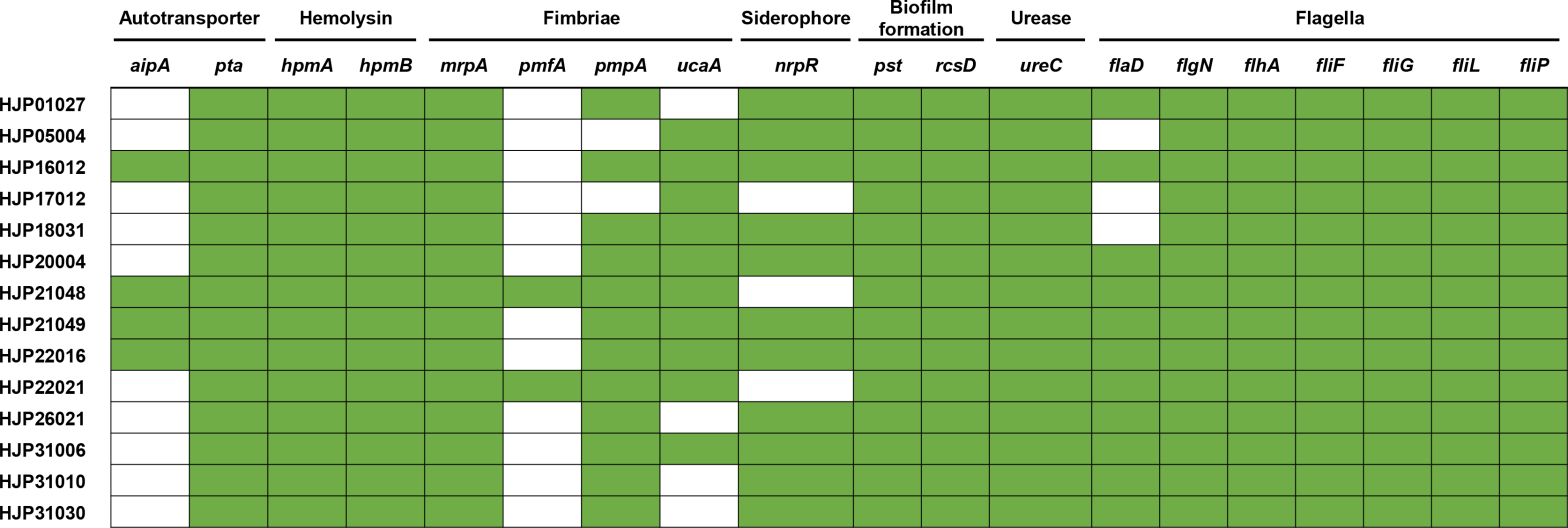
Heatmap of genes related to major bacterial virulence factors. These virulence-associated genes among the isolates are denoted by filled squares (green) for presence and empty squares for absence.

The fimbriae of *P. mirabilis* are essential virulence factors in UTIs (Schaffer et al., 2015). In this study, all the MDR *P. mirabilis* strains contained *mrpA* encoding the MR/P fimbriae, which is known to contribute to biofilm formation and virulence. 85.71% (12/14) strains contained the *pmpA* (encoding the *P. mirabilis* P-like fimbria) and 71.42% (10/14) of them contained the uroepithelial cell adhesin (UCA) fimbriae gene *ucaA*, while only two strains (14.29%) had the *Proteus mirabilis* fimbria (PMF) gene *pmfA* (**Figure 5**). CAUTI is generally initiated by biofilm formation on the urinary catheter (Schaffer et al., 2015). Several factors contribute to *P. mirabilis* biofilm formation *in vitro*, including but not limited to urease, MR/P fimbriae, Pst (phosphate transporter), and RcsD (flagellar regulation) (Schaffer et al., 2015). In addition to *mrpA* as mentioned above, all of the tested strains also contained *ureC* (encoding the urease)*, pst*, and *rcsD* (**Figure 5**). This result provides a possible explanation for the strains’ strong biofilms. It is known that flagella are essential for swimming and swarming motility, which is an important characteristic feature of *P. mirabilis* uropathogenesis (Belas and Suvanasuthi, 2005). Some flagella genes, including *flhA* (encoding the flagellar assembly protein), *fliF* (encoding the flagellar MS-ring protein), *fliG* (encoding the flagellar motor switch protein), *fliP* (encoding the flagellar biosynthetic protein), *fliL* (encoding the flagellar basal body-associated protein), and *flgN* (encoding the flagella filament assembly) were identified in all the tested strains. *flaD* (encoding the flagellar capping protein) was absent in HJP05004, HJP17012, and HJP18031, whose swarming abilities, however, were not affected at all, suggesting that *flaD* maybe not very necessary for swarming motility. As previously reported (Cestari et al., 2013), *hpmAB* (encoding the hemolysin HpmA and its secretion protein HpmB) is highly conserved across the tested *P. mirabilis* isolates, whereas only seven of them exhibited hemolytic activity, suggesting an unknown regulatory mechanism is involved. Besides, 78.57% (11/14) of these *P. mirabilis* strains contained the siderophore synthase gene *nrpR*, which is important for colonizing the urinary tract (Schaffer et al., 2015). It was determined that non-fimbrial adhesins AipA (adhesion and invasion autotransporter) and Pta (*Proteus* toxic agglutinin) are required for invading and colonizing of *P. mirabilis* in the bladder (Armbruster et al., 2018). All of the tested strains carried *pta*, and four (28.57%) of them had *aipA* (**Figure 5**). Taken together, these results demonstrate a number of virulence genes associated with bacterial pathogenicity in these MDR *P. mirabilis* strains, revealing the virulence potential of them.

## 4 Conclusion

In conclusion, this study revealed that ESBL genes are commonly detected in MDR *P. mirabilis* isolates in this hospital, whereas carbapenemase genes are rare. SXT/R391 ICEs, followed by SG1-relative GIs (*Pm*GRI1 in our case) represent major vehicles in mediating the dissemination of ARGs in *P. mirabilis* isolates. Plasmids also contribute to the resistance spread but to a lesser extent. Worryingly, the revelation of virulence-related phenotypes and the presence of abundant virulence genes in these MDR *P. mirabilis* strains pose an urgent threat to public health. Therefore, to better deal with infections caused by this species, close surveillance of the prevalence of MDR strains and related mobile elements from clinical is urgently recommended.

## Data availability statement

The datasets presented in this study can be found in online repositories. The names of the repository/repositories and accession number(s) can be found in the article/**Supplementary Material**.

## Author contributions

YL: conceptualization, formal analysis and writing-original draft. MY, CF and YF: methodology, resources and formal analysis. XD: software. WZ: resources, writing-review and editing. LZ: conceptualization, writing-review and editing, and supervision. All authors contributed to the article and approved the submitted version.
