# Supplementary material for: Genetic analysis of resistance and virulence characteristics of clinical multidrug-resistant Proteus mirabilis isolates
Source: Front Cell Infect Microbiol. 2023 Aug 11;13:1229194. doi: 10.3389/fcimb.2023.1229194 (PMC10457174; doi:10.3389/fcimb.2023.1229194)
Supplement: Supplementary file 1 [file DataSheet_1.docx]

Supplementary Material

Genetic analysis of resistance and virulence characteristics of clinical multidrug-resistant *P. mirabilis* isolates

Ying Li^1^ , Ming Yin^1^, Chengju Fang^1^, Yu Fu^1^, Xiaoyi Dai^1^, Wei Zeng^2,*^, Luhua Zhang^1,*^

^1^The School of Basic Medical Science and Public Center of Experimental Technology, Southwest Medical University, Luzhou 646000, Sichuan Province, China

^2^ Department of Clinical Laboratory, The Hejiang People's hospital, Luzhou 646000, Sichuan Province, China

*** Correspondence:** Luhua Zhang, [zhluhua@swmu.edu.cn](mailto:zhluhua@swmu.edu.cn), or Wei Zeng, mercedes600@126.com.

**Table S1 General features of the genomes of *P. mirabilis* strains.**

| **Strain** | **Draft genome size (bp)** | **GC content** | **No. of contigs** | **No. of contigs ≥ 1,000 bp** | **GenBank**  **accession no.** |
| --- | --- | --- | --- | --- | --- |
| HJP21048 | 3,971,515 | 38.91 | 53 | 45 | JASKYB000000000 |
| HJP21049 | 4,147,345 | 39.08 | 77 | 62 | JASKYD000000000 |
| HJP16012 | 3,973,147 | 38.83 | 44 | 36 | JASKYA000000000 |
| HJP22021 | 4,014,628 | 38.80 | 50 | 44 | JASKYG000000000 |
| HJP05004 | 4,171,159 | 38.95 | 64 | 51 | JASKXX000000000 |
| HJP18031 | 3,964,688 | 38.74 | 40 | 34 | JASKXY000000000 |
| HJP17012 | 4,047,759 | 38.97 | 56 | 47 | JASKXZ000000000 |
| HJP20004 | 4,174,748 | 39.09 | 57 | 46 | JASKYC000000000 |
| HJP31006 | 4,168,404 | 38.92 | 58 | 51 | JASKYH000000000 |
| HJP22016 | 4,250,037 | 38.92 | 83 | 50 | JASKYE000000000 |
| HJP26021 | 4,062,598 | 38.97 | 56 | 46 | JASKYF000000000 |
| HJP31010 | 4,212,070 | 39.00 | 64 | 44 | JASKYI000000000 |
| HJP31030 | 4,056,698 | 38.97 | 55 | 46 | JASKYJ000000000 |
| HJP01027 | 4,066,038 | 38.97 | 54 | 46 | JASKXW000000000 |

**Table S2 Detailed information of *P. mirabilis* strains (from the literature) used in the phylogenetic tree.**

| **Name** | **Source** | **Locations of China** | **Year** | **Accession no.** |
| --- | --- | --- | --- | --- |
| BC11-24 | Animals | Sichuan | 2016 | CP026571 |
| C55 | Animals | Shandong | 2018 | CP044436 |
| JPM24 | Animals | Guangdong | 2017 | CP053894 |
| PmSC1111 | Animals | Sichuan | 2017 | CP034090 |
| RGF134-1 | Animals | Jiangsu | 2019 | CP066833 |
| SNYG35 | Animals | Sichuan | 2018 | CP047589 |
| T21 | Humans | Zhejiang | 2013 | CP017082 |
| XH1653 | Humans | Zhejiang | 2015 | CP065039 |
| YPM35 | Animals | Guangdong | 2017 | CP053898 |
| FZP3320 | Humans | Sichuan | 2021 | JAMOJM000000000 |
| FZP2833 | Humans | Sichuan | 2021 | JAMOJJ000000000 |
| FZP2056 | Humans | Sichuan | 2021 | JAMOBP000000000 |
| FZP2936 | Humans | Sichuan | 2021 | CP098447 |
| FZP3105 | Humans | Sichuan | 2021 | CP098444 |
| FZP4280 | Humans | Sichuan | 2021 | JAMOJR000000000 |
| FZP3725 | Humans | Sichuan | 2021 | JAMOJP000000000 |
| FZP3406 | Humans | Sichuan | 2021 | JAMOJO000000000 |
| JZ12 | Food | Sichuan | 2022 | JAMSBX010000010 |
| JZ17 | Food | Sichuan | 2022 | JAMSBS010000010 |
| JZ18 | Food | Sichuan | 2022 | JAMSBR010000010 |
| JZ19 | Food | Sichuan | 2022 | JAMSBQ010000100 |
| JZ35 | Food | Sichuan | 2022 | JAMSBA010000010 |
| JZ45 | Food | Sichuan | 2022 | JAMSAQ010000010 |
| JZ48 | Food | Sichuan | 2022 | JAMSAO010000010 |
| JZ52 | Food | Sichuan | 2022 | JAMSAJ010000100 |
| JZ53 | Food | Sichuan | 2022 | JAMSAI010000010 |
| JZ56 | Food | Sichuan | 2022 | JAMSAG010000010 |
| JZ66 | Food | Sichuan | 2022 | JAMRZW010000010 |
| JZ96 | Food | Sichuan | 2022 | JAMRZC010000010 |
| STP3 | Animal | Sichuan | 2019 | CP051260 |
| JZ33 | Food | Sichuan | 2022 | JAMSBC010000010 |
| PM1-1 | Animal | Zhejiang | 2021 | JAMKOF010000001 |
| PM1-4 | Animal | Zhejiang | 2021 | JAMKOC010000001 |
| PM1-9 | Animal | Zhejiang | 2021 | JAMKNX010000001 |
| PM2-1 | Animal | Zhejiang | 2021 | JAMKNW010000001 |
| PM2-3 | Animal | Zhejiang | 2021 | JAMKNU010000001 |
| PM2-8 | Animal | Zhejiang | 2021 | JAMKNP010000001 |
| PM3-1 | Animal | Zhejiang | 2021 | JAMKNM010000001 |
| PM3-7 | Animal | Zhejiang | 2021 | JAMKNG010000001 |
| PmBJ012-2 | Humans | Beijing | 2017 | CP065148 |
| PmBJ015-2 | Humans | Beijing | 2017 | CP065147 |
| PmBJ020-1 | Humans | Beijing | 2017 | CP065146 |
| ChSC1905 | Animal | Sichuan | 2019 | CP047929 |


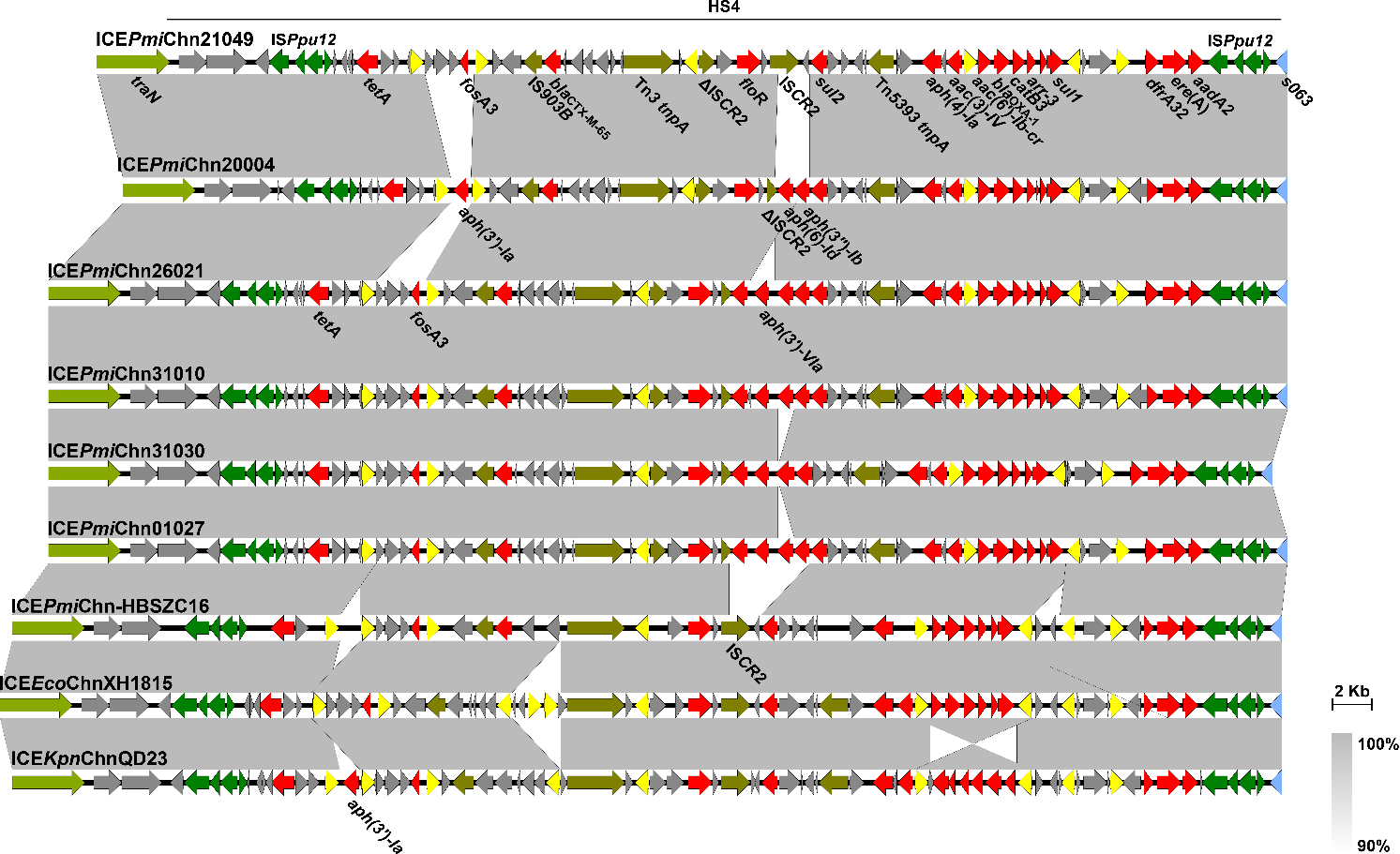


**Figure S1.** **Genetic features of the ICEs in MDR *P. mirabilis* strains.** Genetic characteristics of HS4 and comparisons to those in ICE*Pmi*Chn-HBSZC16 (Accession no. MZ277866), ICE*Eco*ChnXH1815 (CP069386), and ICE*Kpn*ChnQD23(CP042858). ARGs, insertion sequences and restrictive modification (RM) systems are highlighted in red, olive and blue-green, respectively, except that IS*26* is highlighted in yellow. Other genes in the insertion regions are indicated by light blue arrows. Δ represents truncated genes.


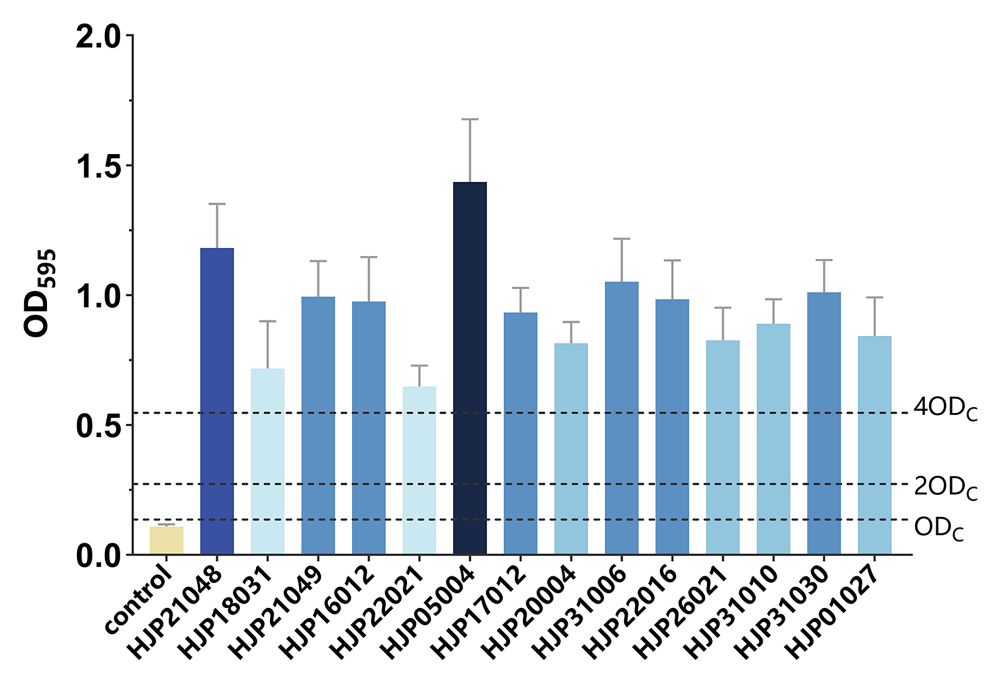


**Figure S2 Crystalline biofilm assay of *P. mirabilis* strains.** The cut-off value (ODc) was defined as 3 SD above the mean OD595 of the negative control. If OD595≤ODc, absence of biofilm; if ODc≤OD595≤2×ODc, weak biofilm producer; if 2×ODc≤OD595≤4×ODc, moderate biofilm producer; if 4×ODc≤OD595, strong biofilm producer.


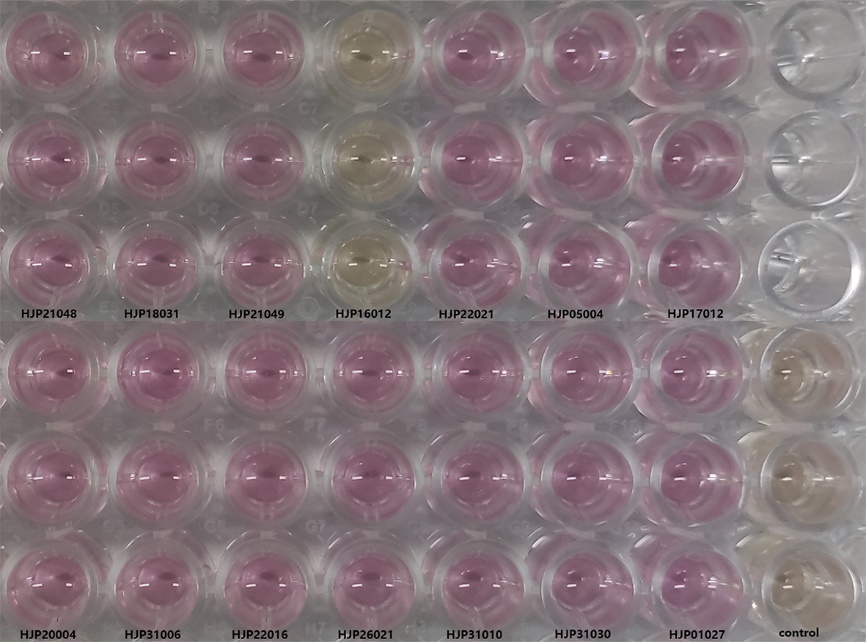


**Figure S3 Urease quantification assay of *P. mirabilis* strains.** Overnight culture of *P. mirabilis* was mixed with LB broth containing filter sterilized urea (20 g/L) in the ratio of 1:100. After incubation for 24 h at 37℃, the color change (orange to pink) was observed by adding 0.02% phenol red reagent (pH indicator).


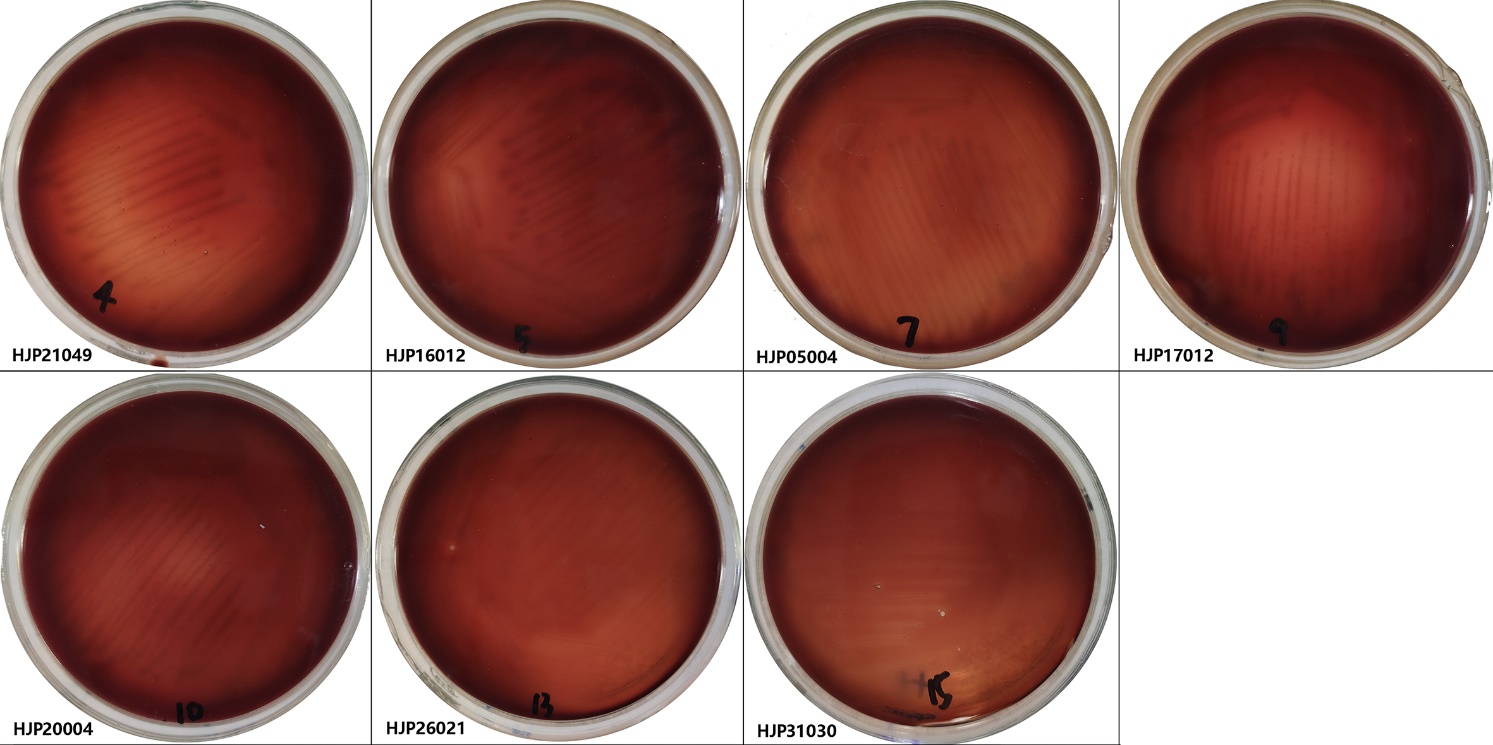


**Figure S4 Hemolysis assay of *P. mirabilis* strains.** The hemolytic ability of *P. mirabilis* strains were determined by culturing on 10% sheep blood plate for 24h at 37℃.


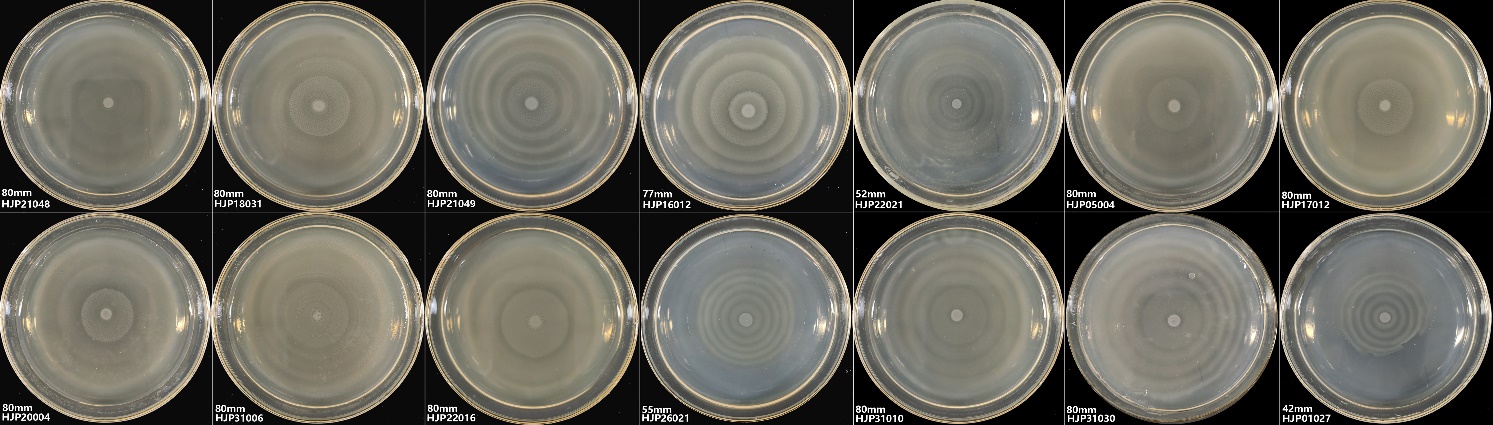


**Figure S5 Motility assay of *P. mirabilis* strains.** 1 µl of culture were point inoculated on to the surface center of the solid LB agar plates. After incubation for 24 h at 37℃ in a lid-side-up position, motility was measured as the diameter across which *P. mirabilis* grew.
